# Magnetic Resonance Imaging Evaluation of Carcinoma of the Cervix With Histopathological Correlation in a Tertiary Care Center: Emphasizing the Rise of Adenocarcinoma in the Indian Context

**DOI:** 10.7759/cureus.71820

**Published:** 2024-10-18

**Authors:** Sanjeev Kishore Subbarayan, Deepu R, Prabu Dhanasingh

**Affiliations:** 1 Radiodiagnosis, Department of Radiology, Karpaga Vinayaga Institute of Medical Sciences and Research Centre, Maduranthakam, IND

**Keywords:** adenocarcinoma, cervical cancer, cervical cancer screening, histopathology, hpv, mri, squamous cell carcinoma

## Abstract

Background and objectives

Adenocarcinoma is increasingly being described in the histological profile of cervical cancer, which is a significant alteration in the disease's profile. This study, which highlights the rising incidence of adenocarcinoma in India, attempts to evaluate the relationship between magnetic resonance imaging (MRI) and histological findings in cervical cancer. The goal is to assess the utility of MRI in tumor staging and its consequences for the management of this evolving histological trend.

Methods

Forty-five patients with cervical cancer were included in a retrospective analysis; they had MRI scans and then histological examinations. We compared the MRI results for tumor size, lymph node involvement, and local invasion with histology, the gold standard for diagnosis. Correlation was evaluated using statistical analysis, which included chi-square tests; a p-value of less than 0.05 was considered significant.

Results

Magnetic resonance imaging showed good agreement with histology (p = 0.03) and was accurate in detecting lesions restricted to the cervix and parametrial infiltration. On the other hand, there were differences in the diagnosis of pelvic lymphadenopathy, which MRI underreported. Magnetic resonance imaging sensitivity was high for detecting parametrial invasion (88.24%) and for tumor extent (81.82%), but lower for lymph node involvement (56.25%). Compared to 47.06% of squamous cell carcinoma (SCC) cases, the study found a considerable increase in the number of instances of adenocarcinoma, with 54.55% of cases presenting in advanced stages. This pattern became statistically significant (p < 0.05), emphasizing the tendency of adenocarcinomas to manifest in their later stages.

Conclusions

When it comes to evaluating local tumor invasion, MRI is a dependable method for staging cervical cancer. However, other diagnostic techniques are required due to their limits in detecting lymphadenopathy. The need for better screening methods specific to this subtype is highlighted by the rising incidence of adenocarcinoma and its propensity for late-stage diagnosis. This will enable early detection and intervention.

## Introduction

In India, cervical cancer is a significant public health concern, accounting for about 96,922 new cases per year [1]. Although squamous cell carcinoma (SCC) has historically been the most common subtype, adenocarcinoma incidence has been gradually increasing, particularly in places like India [2]. Differential patterns of human papillomavirus (HPV) infection, vaccination, and other lifestyle factors such as delayed childbearing and oral contraceptive use are partially responsible for this shift [3]. Since adenocarcinoma is located higher in the endocervical canal than SCC, it is less accessible for standard pap smear screening, which tends to give it a more aggressive course and later diagnosis [4].

Cervical cancer ranks as the second most prevalent gynecologic cancer among women. In contrast to other gynecologic malignancies, cervical cancer tends to manifest in a younger demographic. Early-stage cervical cancers typically exhibit no discernible symptoms, while more advanced stages may present with symptoms such as bleeding, watery discharge, and signs of compression affecting veins, lymphatic vessels, nerves, or ureters [5]. Human papillomavirus is a predominant etiological factor in cervical cancer, implicated in approximately 80%-90% of cases [6]. Cervical carcinoma is histologically classified into two distinct types: SCC and adenocarcinoma. Adenocarcinoma comprises various subtypes, including endometrioid carcinoma, mucinous carcinoma, and mesonephric carcinoma. Approximately 75% of cervical cancer cases are attributed to SCC. Other histological variants, including adenocarcinoma and adenosquamous cell carcinoma, constitute 10%-15% of cases, while other subtypes make up the remaining percentage [7].

Cervical cancer typically exhibits a slow growth pattern, with initial progression occurring laterally along the parametrium and uterosacral ligaments. The neoplasm can extend downward into the vagina and laterally into the paracervical space, potentially invading the urinary bladder, rectum, para-aortic nodes, and pelvic sidewalls in advanced stages or cases of larger tumors [8]. With advancements in medical imaging technology, there has been a significant shift toward non-invasive techniques, with magnetic resonance imaging (MRI) emerging as a superior diagnostic tool due to its high-resolution imaging and detailed soft tissue contrast. Assessing critical prognostic factors like lesion volume and metastatic lymph node involvement is crucial in staging cervical cancer. Magnetic resonance imaging plays a vital role in distinguishing between early-stage disease (stage IIA) and more advanced disease (stage IIB or beyond). The purpose of this study is to investigate the relationship between MRI and histological results, with a particular emphasis on the rising incidence of adenocarcinoma in India and its treatment and diagnosis implications.

## Materials and methods

This retrospective cross-sectional study was carried out in the Department of Radiodiagnosis, Karpaga Vinayaga Institute of Medical Sciences and Research Centre, Madhuranthagam, Chengalpattu, Tamil Nadu. The study included data from June 2022 to July 2024 and included women aged 18 and up. Patients who were referred for an MRI evaluation (Siemens Magnetom 1.5 Tesla (T), Siemens Healthineers, Erlangen, Germany), diagnosed with cervical cancer, and then had surgery were eligible. Histopathological records were reviewed for correlation.

Patients were advised to abstain from eating for six hours before the examination to lessen small bowel movement artifacts. The standard MRI protocol included pelvis-3 plane T2, T1 axial, short tau inversion recovery (STIR) axial, and diffusion sequences. Post-contrast studies were performed by using 10 mL of gadopentetate dimeglumine injection United States Pharmacopeia (USP). Post-contrast imaging was done only on requisition by the referring clinician. Fat-suppressed T2-weighted imaging (T2WI) images were acquired in the axial plane for abdominal screening, and diffusion-weighted sequences were included in certain cases.

Cervical carcinoma staging was done based on the 2018 International Federation of Gynecology and Obstetrics (FIGO) revised system. The assessment parameters included tumor size, tumor enhancement, extension into the vagina and lower uterine body, parametrial/pelvic sidewall involvement, urinary bladder/rectal wall invasion, and lymph node involvement, and were compared to histopathological examination (HPE), which is the gold standard. Chi-square tests were used to assess the data, with a p-value < 0.05 indicating statistical significance.

## Results

The study comprised 45 women who had been diagnosed with cervical cancer. The MRI results revealed that 44.4% of the patients had lesions restricted to the cervix (Stage IB), 13.3% had invasion into the top two-thirds of the vagina (Stage IIA), and 15.6% had parametrial infiltration (Stage IIB). Pelvic lymphadenopathy was seen in 6.67% of cases, while retroperitoneal lymphadenopathy occurred in 8.89% (Table 1).

**Table 1:** MRI findings of the study group (N=45)

MRI findings	Frequency	Percentage
Mass confined to the cervix	20	44.40%
Lesion into the upper 2/3^rd^ of the vagina	6	13.30%
Parametrial infiltration	7	15.60%
Pelvic lymphadenopathy	3	6.67%
Retroperitoneal lymphadenopathy	4	8.89%
Extension to the pelvic wall	1	2.22%
Rectum invasion	1	2.22%
Bladder invasion	1	2.22%

In histological correlation, MRI showed great sensitivity in detecting masses restricted to the cervix and parametrial infiltration. However, there was a minor difference in detecting pelvic lymphadenopathy, with MRI identifying fewer instances than histology (Table 2).

**Table 2:** MRI vs. histopathology correlation (N=45)

MRI findings	MRI (n=45)	Histopathological examination (HPE) (n=45)
Mass confined to the cervix	20	22
Parametrial infiltration	7	7
Pelvic lymphadenopathy	3	5
Lower 1/3^rd^ vaginal extension	2	3

**Figure 1:**
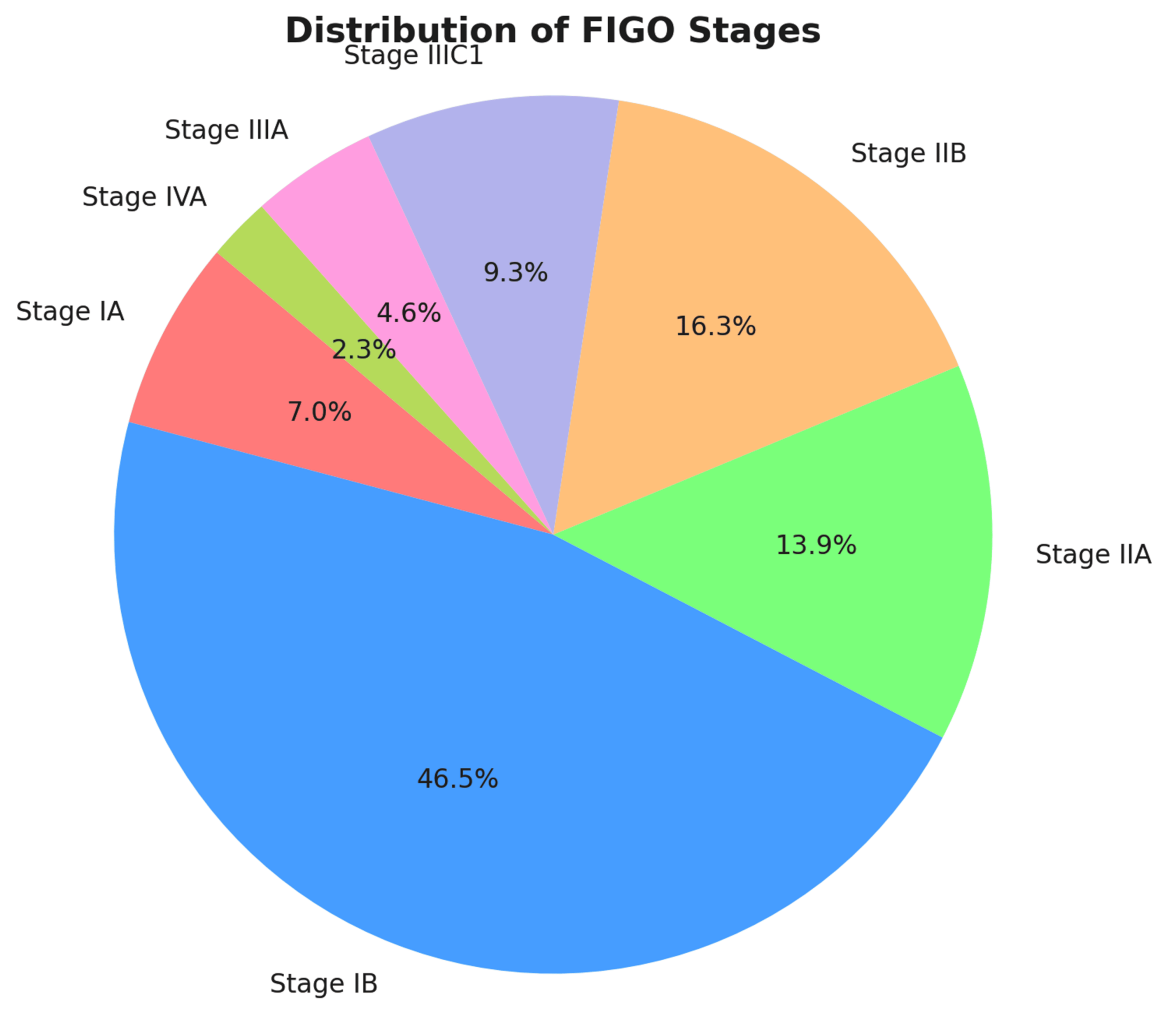
Distribution of cases by FIGO staging (N=45) FIGO: The International Federation of Gynecology and Obstetrics

Magnetic resonance imaging showed high sensitivity for parametric infiltration and lesion confinement to the cervix (p = 0.03). However, there was less consensus about pelvic lymphadenopathy (p = 0.05). The FIGO staging based on MRI results revealed that the majority of patients (44.4%) were in Stage IB, followed by Stage IIA (13.3%) and Stage IIB (15.6%). Advanced stages such as IIIC1 and IVA were uncommon, emphasizing the need for early detection (Figure 1).

**Figure 2:**
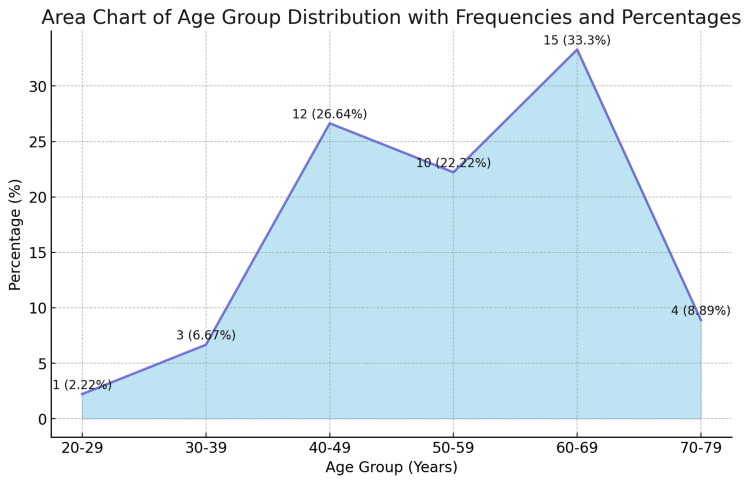
Age-wise distribution of cervical cancer patients (N=45)

The age distribution of patients revealed that the majority were between 50 and 69 years old, with 33.3% falling into the 60-69 age bracket. This demonstrates the increased risk of cervical cancer in postmenopausal women, necessitating improved screening procedures for this age group (Figure 2).

**Figure 3:**
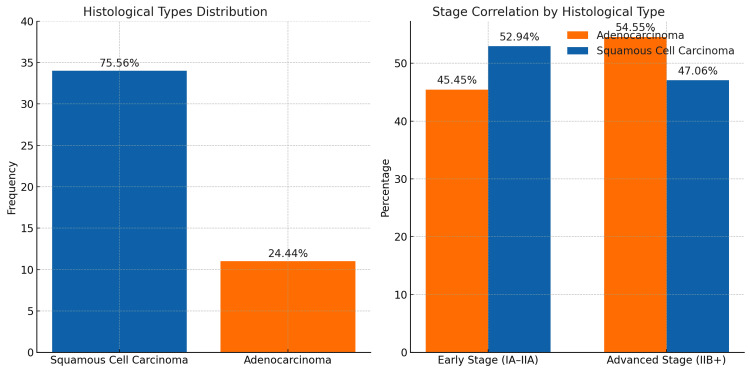
Histological types, distribution, and correlation of adenocarcinoma and squamous cell carcinoma with stages

Squamous cell carcinoma was the predominant histological type in our study, accounting for 75.56% of the cases, while adenocarcinoma constituted 24.44%. The increasing number of adenocarcinoma cases is concerning, given its association with delayed diagnosis and poorer prognosis. Adenocarcinoma cases tend to present at more advanced stages compared to SCC. In our study, 54.55% of adenocarcinoma cases were at an advanced stage (IIB+), while only 47.06% of SCC cases were advanced. This difference, though subtle, reached statistical significance with a p-value < 0.05, indicating a trend toward later-stage detection in adenocarcinoma (Figure 3).

## Discussion

Previous research has demonstrated that MRI is sensitive in detecting lesions restricted to the cervix and parametrial invasion [9,10]. However, the disparities in detecting lymphadenopathy indicate that MRI alone may not be sufficient for nodal staging and should be accompanied by other diagnostic modalities. The rising prevalence of adenocarcinoma in India is alarming, especially as this subtype is frequently detected at a later stage due to its placement in the endocervical canal, making it less accessible to normal pap smears [4]. This conclusion was supported by our investigation, which found that adenocarcinoma cases were more progressed at diagnosis than SCC. The growth in adenocarcinoma is most likely due to changing HPV infection patterns, lifestyle variables, and vaccination trends [3,11].

Human papillomavirus DNA testing and liquid-based cytology may have higher sensitivity for detecting adenocarcinoma, and their incorporation into existing screening programs could boost early detection rates [12,13]. Given the poor prognosis associated with adenocarcinoma, more targeted screening and preventative techniques are required. The distinction between early (IA-IIA) and advanced (IIB and beyond) stages is essential in determining operability and treatment choices, as Stage IIA or lower is generally operable, while Stage IIB often necessitates chemoradiation [14].

In the current study, the comparison between histopathological examination and MRI findings highlights the diagnostic concordance and discrepancies in staging cervical carcinoma. For mass lesions confined to the cervix, MRI correctly identified 20 cases, with two false negatives where the lesions were present but not detected by MRI. This high concordance rate underscores the reliability of MRI in detecting localized cervical lesions. Magnetic resonance imaging showed a perfect match for lesions causing parametrial invasion, lesions extending into the upper two-thirds of the vagina, and lesions extending into pelvic side walls, bladder, and rectal wall infiltration. Pelvic lymphadenopathy was identified in five cases by HPE, with MRI confirming three and missing two cases. This suggests that while MRI is generally reliable for detecting lymph node involvement, additional methods might be needed to improve detection rates. Lesions extending to the lower one-third of the vagina were detected in all two cases by MRI, with HPE detecting three cases, indicating somewhat moderate agreement in identifying lower vaginal involvement. From this study, MRI was found to be very reliable in assessing tumor spread to the parametrium, with a sensitivity of 88.24% for detecting parametrial invasion, 56.25% for detecting lymph node metastasis, and 81.82% sensitivity in determining the total tumor extent. The prognosis may be affected by an underdetection of lymph node involvement due to the decreased sensitivity for lymph node metastasis, which is crucial in deciding if neoadjuvant therapy or surgical resection is the better course of action. The sensitivity values from our study tend to align well with studies by Hricak et al. [15] and their comparison of MRI's diagnostic accuracy against histopathology for parametrial invasion and overall tumor staging, and by Vargas et al. [16], which focused on the sensitivity and specificity of MRI in lymph node metastasis detection in cervical cancer cases.

**Figure 4:**
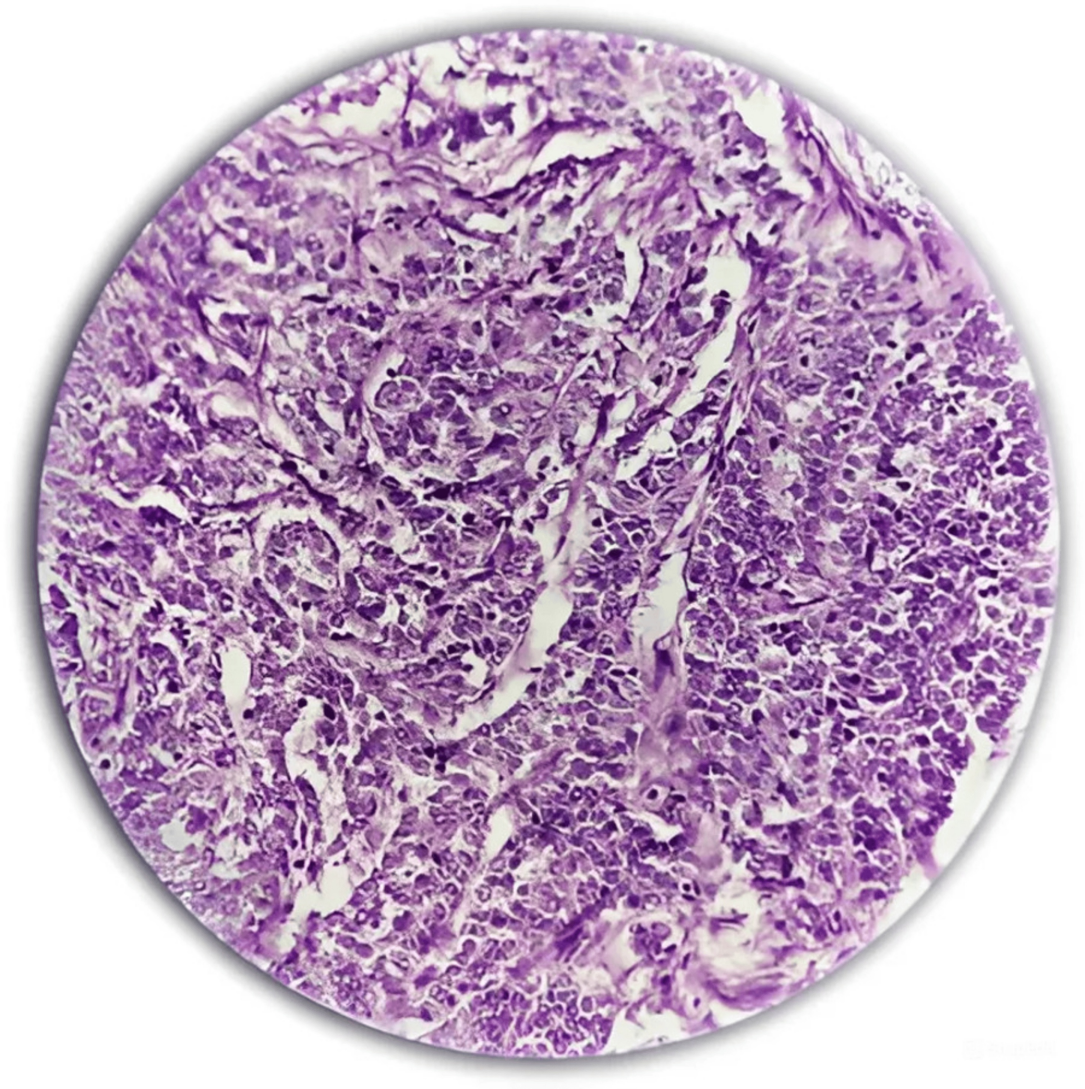
Photomicrograph from a surgical specimen proven histologically to be adenocarcinoma

**Figure 5:**
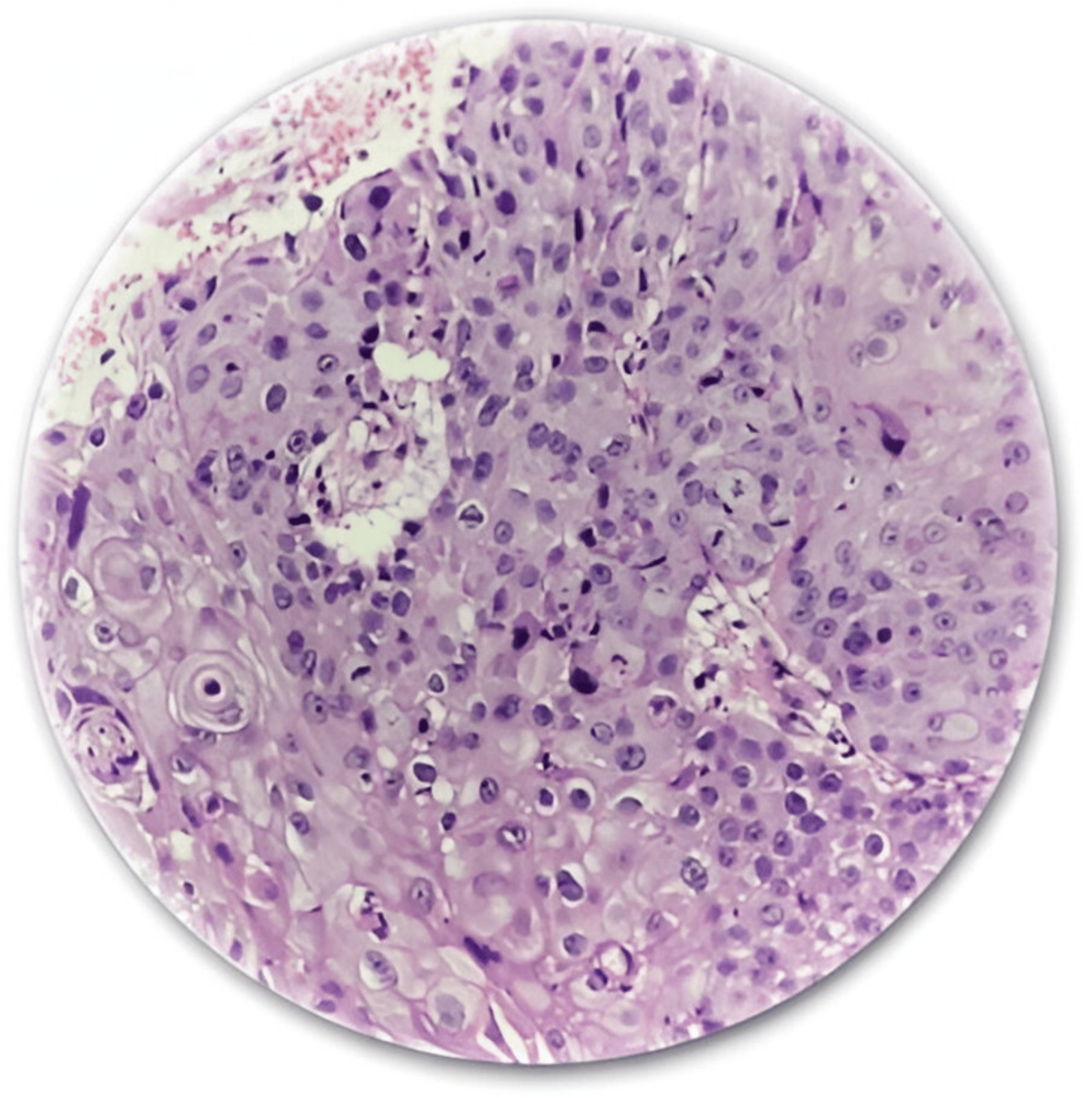
Photomicrograph from a surgical specimen proven histologically to be squamous cell carcinoma

**Figure 6:**
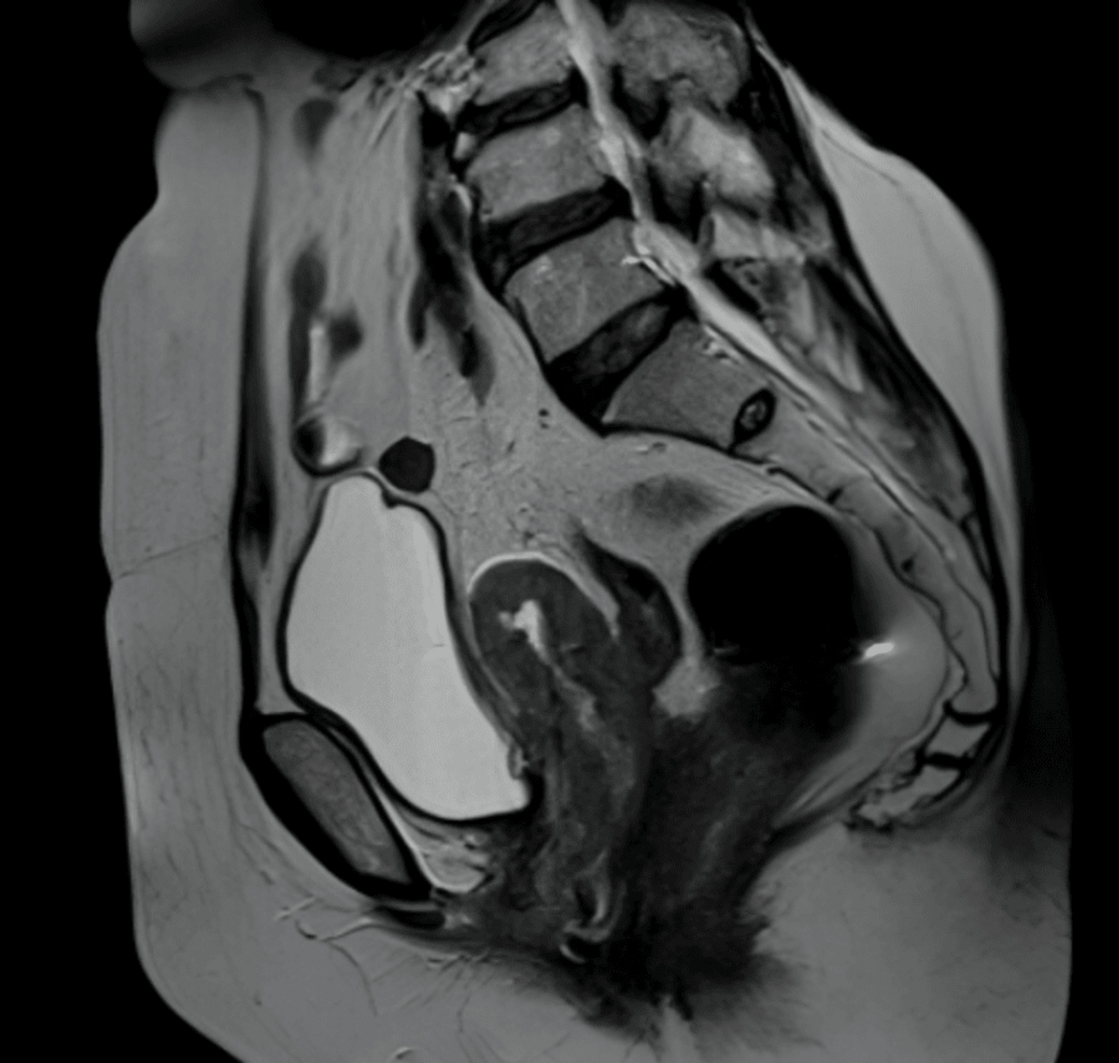
T2-weighted imaging MRI (sagittal view) showing rectal and upper vaginal invasion by cervical carcinoma

**Figure 7:**
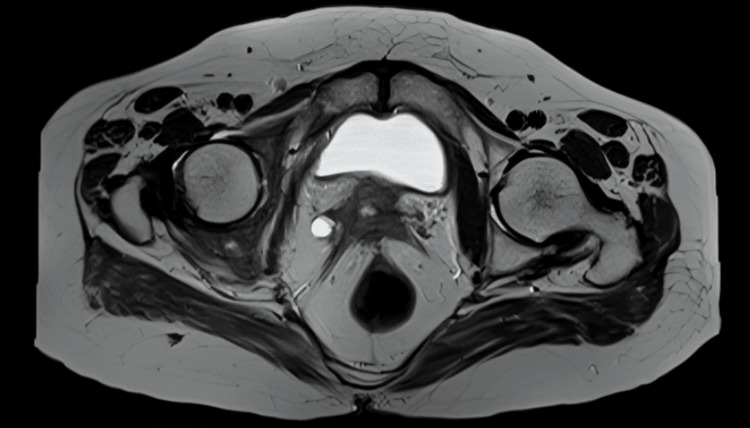
T2-weighted imaging MRI (axial view) demonstrating parametrial invasion and right ureteric invasion with consequent right hydroureteronephrosis

The following images are representative photomicrographs of histopathological sections from the postoperative specimen and preoperative MRI with two examples of locally advanced disease with rectal and right ureteric invasions, respectively, diagnosed on MRI (Figures 4-7).

Limitations

The findings are constrained in their applicability due to the limited sample size and the study's focus on a single center. The retrospective nature introduces potential biases, and variability in imaging equipment and radiologist experience affects the results. Discrepancies between histopathological examination and MRI highlight limitations in both methods, with potential for human error. The wide range of carcinoma stages complicates the assessment of MRI’s accuracy. False negatives and positives indicate MRI is not infallible. Additionally, it does not account for ethnic and genetic variability, which could influence disease progression and MRI accuracy. Addressing these limitations in future research will improve understanding of MRI’s role in diagnosing cervical carcinoma.

## Conclusions

A developing challenge in India is the increasing occurrence of cervical adenocarcinoma. Adenocarcinoma is more likely to be found at an advanced stage and is less susceptible to early detection by pap smear tests than SCC. The varied patterns in HPV vaccination, particularly targeting HPV 18, and lifestyle factors such as delayed childbearing and oral contraceptive use may be contributing to this trend. Because of its higher soft-tissue contrast and sophisticated imaging methods, MRI is a vital diagnostic and staging tool for carcinomas of the cervix. However, limitations in detecting lymph node involvement indicate the need for adjunct imaging or biopsy techniques and therefore it is essential to use histopathological correlation to support diagnosis and guide treatment plans. An immediate assessment of current screening techniques is necessary to ensure early detection and improved outcomes for Indian patients with adenocarcinoma. Ongoing attempts to change screening and imaging protocols are essential.
